# Editorial: Integrative exercise endocrinology

**DOI:** 10.3389/fendo.2023.1350462

**Published:** 2024-01-09

**Authors:** Katarina Tomljenoviċ Borer, Mary Jane De Sousa, Bradley C. Nindl, Kristin I. Stanford, Bente Klarlund Pedersen

**Affiliations:** ^1^ Department of Movement Science, School of Kinesiology, The University of Michigan, Ann Arbor, MI, United States; ^2^ Department of Kinesiology and Physiology, College of Health and Human Development, The Pennsylvania State University, University, Park, PA, United States; ^3^ Warrior Human Performance Research Center, School of Health and Rehabilitation Sciences, University of Pittsburgh, Pittsburgh, PA, United States; ^4^ Department of Surgery, General and Gastrointestinal Surgery, Davis Heart and Lung Research Institute, The Ohio State University College of Medicine, Columbus, OH, United States; ^5^ Centre of Inflammation and Metabolism/Centre for Physical Activity Research, University of Copenhagen, Copenhagen, Denmark

**Keywords:** adipokines, cytokines, exosomes, hepatokines, hormones, myokines, osteokines

Defining exercise endocrinology is not easy largely because of the many different avenues of inter-organ messaging that exercise elicits in its control of metabolism, physiology, behavior, and survival. Although this messaging has been studied for a long time, it continues to change and evolve. The classical view of exercise signaling included autonomic nerves releasing the neurotransmitter norepinephrine and triggering the release of cortisol and adrenal catecholamines (1) for the control of the metabolic fuel mix appropriate for the type, duration, or intensity of exercise (2, 3) or for activation of the life-saving fight-or flight behavioral and physiological responses (4). Hormones, molecules secreted by endocrine glands and released into circulation (5, 6) such as adrenal norepinephrine, epinephrine, and cortisol (1), pituitary growth hormone (GH) (7, 8), IGF (9), and pancreatic glucagon (10) were considered to be the main exercise-associated messengers. The next insight was that exercise could stimulate messaging by paracrine or autocrine means (5, 6) by molecules made in various tissues and organs and acting on other cells and tissues in their vicinity rather than through circulation. Examples are somatostatin in delta pancreatic cells controlling secretion of glucagon from alpha, and of insulin by beta, cells and somatostatin in the stomach inhibiting gastric cells in the antrum (11, 12). Similarly, IGF-gene expression in the muscle is stimulated by mechanical loading to promote *in situ* hypertrophy (13). Realization that exercise-induced changes in hormone pulsatility can affect physiological outcomes, led to the discovery that increased frequency of GH pulses accelerates mature hamster skeletal and somatic growth (14, 15), and that reduced energy availability associated with exercise reduces in female athletes frequency, and increases the amplitude, of LH pulses and abolishes menstrual cycles (16). More recently, explorations of hormone signaling was extended to various body organs which during exercise release messengers into circulation to specific targets. Cytokine messengers like interferon, interleukins, and tumor necrosis factor control immune system and inflammation (17), while insulin-like growth factors control cellular growth (8). Myokines such as irisin, interleukins, and myostatin are released by the muscle (18, 19), hepatokines such as FGF21 and follistatin by the liver (19), adipokines leptin, adiponectin, and resisting are released by the adipose tissue (19, 20). Osteokines like osteocalcin, carboxyterminal propeptide of type-1 collagen (bone formation osteokine), and carboxyterminal peptide of type 1 collagen (bone resorption osteokine) are released from bone osteoblasts and osteoclasts (19, 21, 22). All of these messengers are to a variable extent affected by exercise and play a role in inter-organ communication and actions (19). Finally, exercise also releases bioactive molecules within the extracellular vesicles and exosomes (23, 24).

The editorial team that evaluated the submitted manuscripts was chosen for their expertise in relevant aspects of integrative exercise endocrinology: endocrine changes in the athletes subjected to energy deprivation (25), secretion of exerkines participating in inter-organ communication (26), effects of exercise-induced IGF-1 isoforms in muscle hypertrophy (27), lipokines facilitating muscle lipid metabolism (28), and GH and PTH pulsatility in acceleration of growth (15) and in anabolic responses of postmenopausal bone (22).

Our efforts resulted in publications. Plomgaard et al. presented the regulatory role of glucagon and insulin in the release of hepatokine GDF15. In a clinical study including healthy and anorexic humans, exercise led to increased glucagon to insulin ratio and release of GDF15. Since GDF was also elevated in subjects with anorexia nervosa, this hepatokine may signal chronic energy deprivation. The second manuscript (30) was published by Mohammad et al. describing changes in amyloid-beta precursor protein in an ovariectomized animal. The study with ovariectomized mice demonstrated that voluntary running increased the concentration of an enzyme (BACE1) which limits overproduction of amyloid-beta precursor protein that is implicate in memory loss and Alzheimer disease. The third study was published by Schön et al. about the effects of exercise on growth differentiation factor 11 (GDF11). This cytokine (also called bone morphogenetic protein belonging to TGF alpha family) controls growth, and its gene is found on the chromosome 12. The study reported that an hour of running decreased the concentration of GDF11 in cerebrospinal fluid but not in the blood suggesting cross-talk between the brain and peripheral tissues. The fourth paper was by Hughes et al. presenting an argument that the beneficial increase in bone stiffness arises when the mechanical stimulus of exercise operates during periods of active hormonal influences such as during pubertal growth and administration of PTH analog peritaratide in old age.

This overview of the scope of integrative exercise endocrinology serves, in part, to attract more research in this area of endocrinology and to, hopefully, attract more reports on the Research Topic to this section of Frontiers in Endocrinology.

## Author contributions

KB: Conceptualization, Funding acquisition, Supervision, Writing – original draft, Writing – review & editing. MDS: Validation, Writing – original draft. BN: Writing – original draft, Writing – review & editing. BP: Validation, Writing – original draft, Writing – review & editing. KS: Validation, Writing – original draft, Writing – review & editing.
